# Effects of Smart Goggles Used at Bedtime on Objectively Measured Sleep and Self-Reported Anxiety, Stress, and Relaxation: Pre-Post Pilot Study

**DOI:** 10.2196/58461

**Published:** 2025-01-03

**Authors:** Sharon Danoff-Burg, Elie Gottlieb, Morgan A Weaver, Kiara C Carmon, Duvia Lara Ledesma, Holly M Rus

**Affiliations:** 1 Scientific Research, SleepScore Labs, 2175 Salk Avenue, Suite 150, Carlsbad, CA, 92008, United States, 1 858-299-8995

**Keywords:** relaxation, stress, anxiety, sleep, health technology, intervention

## Abstract

**Background:**

Insufficient sleep is a problem affecting millions. Poor sleep can trigger or worsen anxiety; conversely, anxiety can lead to or exacerbate poor sleep. Advances in innovative consumer products designed to promote relaxation and support healthy sleep are emerging, and their effectiveness can be evaluated accurately using sleep measurement technologies in the home environment.

**Objective:**

This pilot study examined the effects of smart goggles used at bedtime to deliver gentle, slow vibration to the eyes and temples. The study hypothesized that objective sleep, perceived sleep, self-reported stress, anxiety, relaxation, and sleepiness would improve after using the smart goggles.

**Methods:**

A within-participants, pre-post study design was implemented. Healthy adults with subclinical threshold sleep problems (N=20) tracked their sleep nightly using a polysomnography-validated noncontact biomotion device and completed daily questionnaires over two phases: a 3-week baseline period and a 3-week intervention period. During the baseline period, participants followed their usual sleep routines at home. During the intervention period, participants used Therabody SmartGoggles in “Sleep” mode at bedtime. This mode, designed for relaxation, delivers a gentle eye and temple massage through the inflation of internal compartments to create a kneading sensation combined with vibrating motors. Each night, the participants completed questionnaires assessing relaxation, stress, anxiety, and sleepiness immediately before and after using the goggles. Daily morning questionnaires assessed perceived sleep, complementing the objective sleep data measured every night.

**Results:**

Multilevel regression analysis of 676 nights of objective sleep parameters showed improvements during nights when the goggles were used compared to the baseline period. Key findings include sleep duration (increased by 12 minutes, *P*=.01); duration of deep sleep (increased by 6 minutes, *P*=.002); proportion of deep sleep (7% relative increase, *P*=.02); BodyScore, an age- and gender-normalized measure of deep sleep (4% increase, *P*=.002); number of nighttime awakenings (7% decrease, *P*=.02); total time awake after sleep onset (reduced by 6 minutes, *P*=.047); and SleepScore, a measure of overall sleep quality (3% increase, *P*=.02). Questionnaire responses showed that compared to baseline, participants felt they had better sleep quality (*P<*.001) and woke feeling more well-rested (*P<*.001). Additionally, participants reported feeling sleepier, less stressed, less anxious, and more relaxed (all *P* values <.05) immediately after using the goggles each night, compared to immediately before use. A standardized inventory administered before and after the 3-week intervention period indicated reduced anxiety (*P*=.03), confirming the nightly analysis.

**Conclusions:**

The use of smart goggles at bedtime significantly improved objectively measured sleep metrics and perceived sleep quality. Further, participants reported increased feelings of relaxation along with reduced stress and anxiety. Future research expanding on this pilot study is warranted to confirm and expand on the preliminary evidence presented in this brief report.

## Introduction

Insufficient sleep affects approximately one-third of the population [1] and is associated with adverse health outcomes and impaired performance [2]. Poor sleep can trigger or worsen anxiety, while conversely, anxiety can lead to or exacerbate poor sleep [3]. Technological advances in unobtrusive sleep measurement enable intervention studies to be conducted in the comfort of research participants’ own bedrooms, providing ecologically valid results while capturing accurate objective data [4,5]. Concomitantly, the development of innovative consumer products designed to promote relaxation and support healthy sleep is emerging. Their effectiveness can be evaluated in field studies using ambulatory measurement technologies [6].

The aim of this research was to examine the effects of smart goggles used at bedtime to deliver gentle vibration to the eyes and temples, on sleep as well as on perceived stress, anxiety, and relaxation. Although a variety of evidence-based relaxation techniques [7] and sleep-enhancing products [8] already exist, devices such as smart goggles may appeal to individuals who wish to use such technological tools as an option within their repertoire of strategies for winding down at bedtime. Previous research suggests that vibration can increase relaxation [9], induce drowsiness [10], and may be a useful nonpharmacological intervention for poor sleep [11-13]. For example, a preliminary study documented the use of vibration in improving objectively measured sleep outcomes in people with mild to moderate symptoms of insomnia [11]. In this pilot study on adults with subclinical threshold sleep problems, we hypothesized that objective and perceived sleep outcomes as well as self-reported stress, anxiety, relaxation, and sleepiness would improve after using smart goggles delivering slow vibrations at bedtime.

## Methods

### Participants

Invitations to complete an eligibility questionnaire for a study testing smart goggles were emailed to registrants in a large database of individuals interested in sleep research and using SleepScore technology. Eligible respondents were invited to enroll based on the following selection criteria: difficulty falling or staying asleep, no history of sleep disorders, absence of other medical issues affecting sleep, substance use that could affect sleep, and no lifestyle issues such as shift work that might influence their sleep. The study included adults (N=20) with subclinical threshold sleep problems who were willing to track their sleep and use the smart goggles as instructed.

### Design and Procedures

A within-participants, pre-post study design was implemented. The participants were aware that the intervention had the potential to affect their sleep. Following a 3-week baseline period during which participants measured their sleep data at home without any intervention, they used the Therabody SmartGoggles (Therabody Inc, Los Angeles, CA) at bedtime for 15 minutes (within 30 minutes of their intended sleep time) over a 3-week intervention period. The participants were instructed to use “Sleep” mode, designed for relaxation and inducing sleepiness. This mode delivers slow and gentle massage to the eye and temple areas by the inflation of internal compartments to create a kneading sensation and vibrating motors. Additionally, two other modes (SmartRelax and Focus) are available; however, since these provide different experiences, they were not used in this study.

During the entire 6-week study, nightly objective sleep measurements were collected and participants completed online questionnaires each morning and evening. Data collection was synchronized across all participants to account for weekday or weekend variation.

### Measurement

Objective sleep data were collected with SleepScore Max (Consumer Sleep Solutions LLC, Carlsbad, CA), a noncontact monitoring device that uses respiratory signal and motion sensing to detect sleep. The device is placed next to the bed and controlled using a companion app. It uses ultra-low power radiofrequency waves to monitor body movement and respiration patterns when in bed; the measurement is unaffected by bedding or nightwear. If a partner is present, only the sleep of the individual closest to the device is measured. The device captures high-resolution magnitude and duration data of gross movements, micromovements, and full breathing cycles, which are transformed into 30-second epoch sleep stage data (wake, light, deep, rapid eye movement [REM]) using proprietary algorithms. Studies have shown good agreement with gold-standard polysomnography [14,15], exceeding the accuracy typically reported for actigraphy-based devices [16].

Using the 30-second epoch data, standard sleep metrics were calculated. In addition, 3 SleepScore technology proprietary sleep metrics reflecting sleep quality, all ranging from 0 to 100 and normalized for age and gender using reference values from the meta-analysis of quantitative sleep parameters by Ohayon and colleagues [17], were calculated:

- *SleepScore* is an overall sleep quality metric that includes objectively measured total sleep time, sleep onset latency, and sleep stage durations.
- *BodyScore* reflects the age- and gender-normalized amount of deep (non–rapid eye movement stage 3 [NREM-3]) sleep.
- *MindScore* reflects the age- and gender-normalized amount of REM sleep.

Self-reported data were collected daily, across the entire study via 100-point visual analog scales. Morning assessments measured perceived sleep quality and feeling well-rested upon waking. At night, the 100-point visual analog scales assessed relaxation, stress, anxiety, and sleepiness before and after goggle use. The construct validity and discriminate sensitivity of visual analog scales to assess perceived stress and related constructs have been documented [18]. Participants completed these scales immediately before using the goggles at bedtime and after using the goggles for 15 minutes, and then went to sleep. A 6-item version of the state scale of the Spielberger State-Trait Anxiety Inventory (STAI) [19] was administered once before and once after the intervention period.

### Statistical Analyses

Nightly objective sleep data and daily self-reported data were analyzed using multilevel regression with random intercept, accounting for nights nested within participants, comparing nights during the baseline period to nights during the intervention period for each outcome. The regression model was Sleepmeasure_ij_ = β_0_ + β_1_*TestPeriod_ij_ + u_0j_ + e_ij_; TestPeriod, coded as 0 for observations during baseline and 1 for nights during the intervention period. Similarly, analysis of the nightly self-reported data used the same model to compare pre- and post-goggle use. A 2-tailed paired-samples *t* test was used to analyze changes in the 6-item STAI scores.

Discrepancies in sample sizes (N=20 for objective and self-reported sleep, n=17 for 6-item STAI) were due to incomplete data sources. Participants tracked their sleep at home and, at times, were not fully compliant with the use of measurement tools or the completion of online surveys. This is common in field research collecting longitudinal and daily assessment data. All reported results reflect the largest available sample for each set of analyses.

### Ethical Considerations

The study was approved by Sterling Institutional Review Board (ID 11012), and all procedures were conducted in accordance with the ethical standards of the Declaration of Helsinki. All participants provided informed consent using a secure online platform to review, electronically sign, and return a copy of the document to the research coordinator. They were informed that the study was voluntary, and of their right to withdraw at any time. Both objective and self-reported study data were deidentified prior to analysis and accessible only to members of the research team. As compensation, participants kept the smart goggles and sleep measurement device used during the study.

## Results

Of the 20 participants, 40% (n=8) were women, and the age range was 26-75 (mean 50.41, SD 13.12) years. Further demographic details are provided in Table 1.

**Table 1.** Demographic information of participants at baseline (N=20).

Demographics	Values
Age (years)	
Mean (SD)	50.41 (13.12)
Range	26‐75
Gender, n (%)	
Men	8 (40)
Women	12 (60)
Other identities	0 (0)
Race/Ethnicity, n (%)	
American Indian or Alaska Native	0 (0)
Asian	1 (5)
Black or African American	0 (0)
Hispanic/Latino	4 (20)
Native Hawaiian or other Pacific Islander	1 (5)
White	14 (70)
Household composition, n (%)	
I live alone	7 (35)
I live with my partner - (currently) no children in the house	3 (15)
I live with my children - (currently) no partner in the house	1 (5)
I live with my partner and child(ren)	6 (30)
I live with a family member(s)	3 (15)
I live with a roommate(s)	0 (0)
Other	0 (0)
Employment, n (%)	
Working full-time	14 (70)
Working part-time	2 (10)
Homemaker	2 (10)
Full-time student	1 (5)
Retired	1 (5)
Education, n (%)	
High school degree or equivalent (eg, GED^a^)	1 (5)
Some college, no degree	5 (25)
Associate degree	1 (5)
Bachelor’s degree	8 (40)
Master’s degree	3 (15)
Doctoral degree	2 (10)
Annual household income (US$), n (%)	
<$25,000	0 (0)
$25,000-$49,999	3 (15)
$50,000-$74,999	4 (20)
$75,000-$99,999	3 (15)
$100,000-$124,999	2 (10)
$125,000-$149,999	2 (10)
$150,000-$174,999	0 (0)
$175,000-$199,999	0 (0)
≥$200,000	5 (25)
Would rather not answer this question	1 (5)

a GED: General Educational Development.

### Objective Sleep

Nightly objective measurement of sleep (n=676 nights nested within 20 participants) revealed multiple improvements when participants used the goggles at bedtime. Key findings included increased sleep duration (+12 min, *P*=.01); increased deep sleep, reflected both in duration (+6 min, *P*=.002) and proportion of the night (7% relative increase, *P*=.02); enhanced BodyScore (+4%, *P*=.002); fewer nighttime awakenings (−7%, *P*=.02); reduced total wake time at night after sleep onset (−6 min, *P*=.047); and improved SleepScore, indicating overall sleep quality (+3%, *P*=.02). Detailed objective sleep metrics are displayed in Table 2.

**Table 2.** Multilevel regression results for objective sleep (n=676 nights nested within 20 participants), comparing the baseline period (sleep at home prior to intervention) to the intervention period (sleep at home using smart goggles at bedtime).

Outcomes	Objective measurement of sleep, observed mean (SD)^a^		Estimated marginal means^b^		
	Baseline period	Intervetion period	Intercept (SE)	*β^c^*	*P* value
Total sleep time in minutes	331.55 (57.49)	343.48 (62.69)	331.52 (4.87)	12.04	.01
Sleep onset latency in minutes	23.34 (18.33)	23.20 (18.17)	23.28 (1.64)	−0.13	.92
Number of awakenings	5.51 (2.10)	5.14 (2.12)	5.51 (0.17)	−0.38	.02
Time spent awake after sleep onset in minutes	60.50 (31.30)	54.32 (31.38)	60.17 (2.72)	−5.39	.047
Light sleep in minutes	215.36 (49.64)	219.86 (50.57)	215.47 (3.99)	4.54	.26
Deep sleep in minutes	57.52 (19.92)	63.19 (24.27)	57.66 (1.79)	5.58	.002
REM^d^ sleep in minutes	58.67 (23.54)	60.44 (25.55)	58.52 (1.93)	1.94	.32
Percentage of time spent awake after sleep onset	16 (7)	14 (7)	15.36 (0.58)	−1.02	.08
Percentage of time in light sleep	54 (8)	55 (8)	54.50 (0.60)	0.02	.97
Percentage of time in deep sleep	15 (6)	16 (7)	15.10 (0.49)	1.14	.02
Percentage of time in REM sleep	15 (6)	15 (6)	14.92 (0.46)	−0.12	.80
SleepScore^e^	69.57 (10.89)	71.65 (10.87)	69.61 (0.87)	2.02	.02
BodyScore^e^	73.14 (10.38)	76.13 (11.15)	73.42 (0.86)	2.64	.002
MindScore^e^	69.30 (13.76)	69.68 (14.48)	69.18 (1.13)	0.55	.62

a For the baseline and intervention periods, each mean was calculated by averaging nights across participants, then averaging those participants’ averages to a single simple average.

b These are the outcomes of separate multilevel regression analyses. Each row shows results from a different single-predictor, single-outcome model.

c The beta values are unstandardized and can therefore be interpreted on the same scale as the original data.

d REM: rapid eye movement.

e These scores range from 0 to 100. SleepScore is an age- and gender-normalized measure of overall sleep quality, BodyScore is an age- and gender-normalized measure of deep sleep, and MindScore is an age- and gender-normalized measure of REM sleep.

### Self-Reported Sleep Quality and Well-Restedness

Multilevel regression analyses of daily self-reported sleep data (N=723 nights nested within 20 participants) showed that participants perceived better sleep quality (*β*=12.37, *P<*.001) and felt more rested in the morning (*β*=12.13*, P<*.001) when using the goggles at bedtime compared to baseline, as assessed on a scale from 0 to 100.

### Sleepiness, Anxiety, Stress, and Relaxation

Across 334 nights of the intervention period, multilevel regression analyses comparing responses immediately after using the goggles to those reported immediately before showed that participants felt sleepier (*β*=9.98, *P<*.001), less stressed (*β*=−10.38*, P<*.001), less anxious (*β*=−12.87*, P<*.001), and more relaxed (*β*=11.76*, P<*.001), all rated on a scale from 0 to 100.

At the end of the intervention period, compared to the end of the baseline period, participants’ scores on the 6-item STAI showed reduced anxiety (*t*_16_=2.31, *P*=.03), reflecting a 10% decrease and confirming the nightly analyses.

## Discussion

Nonpharmacological techniques for promoting relaxation and improving sleep have the potential to help millions of individuals who experience suboptimal sleep [20]. This study evaluated the effectiveness of smart goggles designed to induce relaxation and support healthy sleep. Outcomes were measured using both self-reported data and a polysomnography-validated, noncontact biomotion device. The study population included a nonclinical sample of adults reporting poor sleep in the absence of diagnosed sleep disorders.

Compared to baseline, using the goggles at bedtime led to objective improvements in both sleep quality and duration. Although total sleep time remained less than 6 hours per night on average, objective improvements were seen in several parameters, including:

- sleep duration;
- increased deep sleep, both in duration and as a proportion of the night;
- reduced number of nighttime awakenings;
- decreased time spent awake at night after initially falling asleep; and
- enhanced sleep quality.

Aligning with these objective results, questionnaire data showed that participants perceived improvement in their sleep quality and felt more well-rested in the morning. In addition, immediately after using the goggles, participants felt sleepier, less stressed, less anxious, and more relaxed, compared to their experience immediately before using the goggles. A standardized inventory administered before and after the 3-week intervention period also indicated reduced anxiety, confirming the nightly analysis.

The observed improvements in the objective and self-reported sleep data may be attributed to increased relaxation resulting from the use of the goggles at bedtime. Vibration has been previously shown to be able to induce physiological relaxation [9,10] and support sleep [11-13]. This interpretation is supported by the changes in perceived relaxation, stress, and anxiety. However, objective parameters of physiological relaxation prior to sleep were not assessed, presenting an interesting avenue for future research.

Further studies could explore the intervention through a controlled trial, including comparison to goggles without vibration or to other relaxation techniques. This study assessed changes in parameters from the baseline to the intervention period while using the product at home, resembling how it is used outside of a research setting. The within-participants study design, which included long-term product use following a baseline period without the intervention, provides confidence that significant effects are due to the intervention itself. Although this study design has limitations, it reflects the real-world experience of introducing an intervention into the home environment, while also accounting for night-to-night variations in sleep patterns.

While longitudinal data provides an advantage by enabling the detection of nightly within-person differences, this pilot study is limited by its small sample size. Although we compared participants’ improvements in sleep and related outcomes to their own baseline, the small sample size could have introduced a potential bias, and therefore these results should be interpreted with caution. Future research should investigate the effects of this intervention using larger sample sizes. Another limitation is that contactless technology is not ambulatory and therefore does not capture objective measures of daytime activity. However, the noncontact sleep measurement system used in this study offers the advantage of significantly higher overall accuracy, particularly in specificity, compared to traditional research-based actigraphy [16].

To conclude, this study found that using vibration therapy administered via smart goggles before going to sleep was associated with improvements in both objective sleep measures and self-reported outcomes. While these findings must be interpreted with caution, our data suggest that a device delivering gentle vibrations to the eye and temple area may have the potential to promote relaxation, decrease anxiety, and support healthy sleep when used at bedtime by adults with suboptimal sleep. Not only did sleep quality and sleep duration increase relative to baseline, but there was a decrease in the number of awakenings and the duration of time spent awake during the night. Further, improvement was seen in multiple metrics of deep sleep, which is vital for brain health and physical recovery. Future research expanding on this pilot study is warranted to confirm the preliminary evidence presented in this study.

## Abbreviations

NREM-3: non–rapid eye movement stage 3
REM: rapid eye movement
STAI: State-Trait Anxiety Inventory
